# Reconstruction of the High Stigma Exsertion Rate Trait in Rice by Pyramiding Multiple QTLs

**DOI:** 10.3389/fpls.2022.921700

**Published:** 2022-06-07

**Authors:** Quanya Tan, Suhong Bu, Guodong Chen, Zhenguang Yan, Zengyuan Chang, Haitao Zhu, Weifeng Yang, Penglin Zhan, Shaojun Lin, Liang Xiong, Songliang Chen, Guifu Liu, Zupei Liu, Shaokui Wang, Guiquan Zhang

**Affiliations:** ^1^Guangdong Provincial Key Laboratory of Plant Molecular Breeding, State Key Laboratory for Conservation and Utilization of Subtropical Agro-Bioresources, South China Agricultural University, Guangzhou, China; ^2^Guangdong Laboratory for Lingnan Modern Agriculture, South China Agricultural University, Guangzhou, China

**Keywords:** outcrossing, stigma exsertion, QTL-pyramiding, epistasis, trait reconstruction, rice

## Abstract

Asian cultivated rice is a self-pollinating crop, which has already lost some traits of natural outcrossing in the process of domestication. However, male sterility lines (MSLs) need to have a strong outcrossing ability to produce hybrid seeds by outcrossing with restorer lines of male parents in hybrid rice seed production. Stigma exsertion rate (SER) is a trait related to outcrossing ability. Reconstruction of the high-SER trait is essential in the MSL breeding of rice. In previous studies, we detected eighteen quantitative trait loci (QTLs) for SER from *Oryza sativa, Oryza glaberrima*, and *Oryza glumaepatula* using single-segment substitution lines (SSSLs) in the genetic background of Huajingxian 74 (HJX74). In this study, eleven of the QTLs were used to develop pyramiding lines. A total of 29 pyramiding lines with 2–6 QTLs were developed from 10 SSSLs carrying QTLs for SER in the HJX74 genetic background. The results showed that the SER increased with increasing QTLs in the pyramiding lines. The SER in the lines with 5–6 QTLs was as high as wild rice with strong outcrossing ability. The epistasis of additive by additive interaction between QTLs in the pyramiding lines was less-than-additive or negative effect. One QTL, *qSER3a-sat*, showed minor-effect epistasis and increased higher SER than other QTLs in pyramiding lines. The detection of epistasis of QTLs on SER uncovered the genetic architecture of SER, which provides a basis for using these QTLs to improve SER levels in MSL breeding. The reconstruction of the high-SER trait will help to develop the MSLs with strong outcrossing ability in rice.

## Introduction

Every cultivated crop was once wild. In the process of domestication, crop species increased their productivity and narrowed their genetic base. The result is that many crops contain only a small fraction of the genetic variation of their wild relatives (Zamir, 2001). Asian cultivated rice (*Oryza sativa*) is a self-pollinating crop with <1% natural cross-pollination (Virmani and Athwal, 1973). During domestication, the homozygosity of varieties led to a steady decline in the outcrossing ability of cultivated rice. In contrast, African cultivated rice (*Oryza glaberrima*), which has been domesticated for a shorter period of time, has more wild characteristics in floral traits and outcrossing ability (Marathi and Jena, 2015; Marathi et al., 2015). Wild *Oryza* species were more outcrossed due to their larger stigmas, longer styles, higher stigma exsertion, and longer spikelet opening period, with perennial species being more outcrossed than annual species (Oka and Morishima, 1967; Parmar et al., 1979; Marathi and Jena, 2015; Marathi et al., 2015). Therefore, the cultivated rice has lost some traits of natural outcrossing during domestication (Parmar et al., 1979).

Hybrid rice was successfully applied in China in the 1970s. Since then, hybrid rice has been widely planted in China and around the world (Yuan and Virmani, 1988). The hybrid rice exhibits strong heterosis, which plays an important role in improving rice yield (Cheng et al., 2007; Zhang et al., 2021; Zhang, 2022). Since male sterility lines (MSLs) are used to produce hybrid seeds by outcrossing with restorer lines of male parents, the outcrossing ability of MSLs is a key factor in improving hybrid seed yield. Therefore, the improvement of outcrossing ability is an important goal of MSL breeding. Stigma exsertion rate (SER) is an evaluating indicator of outcrossing ability and is controlled by quantitative trait loci (QTLs). Over the past decades, many QTLs controlling SER and related traits from different genetic resources have been located in the rice genome (Marathi and Jena, 2015; Zhou et al., 2017a; Liu et al., 2019; Tan et al., 2022). Some of the QTLs were detected in relative species of the cultivated rice, such as *O. glaberrima* (Tan et al., 2022), *Oryza rufipogon* (Xiong et al., 1999; Li et al., 2001; Uga et al., 2003a; Huang et al., 2012; Bakti and Tanaka, 2019; Zou et al., 2020), *Oryza barthii* and *Oryza meridionalis* (Zou et al., 2020), *Oryza longistaminata* (Li et al., 2010), and *Oryza glumaepatula* (Tan et al., 2020). The detection of QTLs for SER laid a foundation for the improvement of outcrossing ability of MSLs.

Understanding the genetic architecture of traits is a prerequisite for trait reconstruction. Genetic architecture of quantitative traits includes the numbers and genome locations of genes affecting a trait, the magnitude of their effects, and the relative contributions of additive, dominant, and epistatic gene effects (Kroymann and Mitchell-Olds, 2005; Holland, 2007). Epistasis is defined as interactions between genome-wide loci, impacting target traits either additively increasing each other's phenotypic effects or non-additively counteracting the effects of the major loci, *via* other loci that interact epistatically (Mackay, 2014; Misra et al., 2021). Epistasis complicates the genotype–phenotype relationship because it causes hidden quantitative genetic variation in natural populations and may lead to the small additive effects (Holland, 2007; Mackay, 2014). The role of epistasis in the genetic architecture of quantitative traits is controversial due to the fact that most genetic variation for quantitative traits is additive (Mackay, 2014). In QTL mapping approaches, first-order effects are commonly fitted before second- and higher-order (epistatic) effects, which makes it statistically difficult to detect epistatic effects (Gaertner et al., 2012; Doust et al., 2014). The studies in *Arabidopsis* and rice suggested that epistatic QTL effects were more important than additive QTL for fitness traits (Malmberg et al., 2005; Mei et al., 2005). By contrast, the studies designed to explicitly model epistatic interactions in maize revealed that epistasis was of less or only moderate importance for quantitative traits (Schon et al., 2004; Mihaljevic et al., 2005; Blanc et al., 2006; Holland, 2007).

Like near isogenic lines (NILs), single-segment substitution lines (SSSLs) carry only one chromosome substitution segment from donors in a recipient genetic background (Zhang et al., 2004; Keurentjes et al., 2007; Zhang, 2021). We have developed an SSSL library, which includes 2,360 SSSLs derived from 43 donors of 7 species of rice AA genome in the Huajingxian 74 (HJX74) genetic background (Zhang et al., 2004; Xi et al., 2006; Zhang, 2021). The HJX74-SSSLs were widely used to detect QTLs for traits of agronomic importance (Zhang et al., 2012; Zhu et al., 2014, 2018; Yang et al., 2016, 2021a,b; Zhou et al., 2017b; Pan et al., 2021; Zhan et al., 2021; Fu et al., 2022), to clone genes of functional importance, and to mine alleles of functional variants (Zeng et al., 2006; Teng et al., 2012; Wang et al., 2012; Fang et al., 2019; Sui et al., 2019; Gao et al., 2021; Zhan et al., 2022). Using the HJX74-SSSL library as a platform for rice design, a series of cytoplasmic male sterility (CMS), maintainer and restorer lines, and wide-compatible *indica* lines (WCILs) were developed (Dai et al., 2015, 2016; Luan et al., 2019; Guo et al., 2022). Recently, the SSSLs were used to detect QTLs controlling SER in rice. Eighteen QTLs for SER from *O*. *sativa, O*. *glaberrima*, and *O*. *glumaepatula* were detected in the HJX74-SSSLs (Tan et al., 2020, 2021, 2022). In this study, eleven of the QTLs were used to develop 2- to 6-QTL pyramiding lines in the HJX74 genetic background. The results showed that the SER increased with increasing QTLs in the pyramiding lines. The epistasis of additive by additive interaction between QTLs in 2- to 6-QTL lines was less-than-additive or negative effect. The pyramiding lines carrying 5–6 QTLs for SER showed as high SER as wild rice. The reconstruction of the high SER trait will help to develop MSLs with strong outcrossing ability in rice.

## Materials and Methods

### Plant Materials

In total, eleven of the QTLs for SER were used to develop QTL-pyramiding lines. Among the 11 QTLs, four were from *O*. *sativa* (Tan et al., 2021), four were from *O*. *glaberrima* (Tan et al., 2022), and three were from *O*. *glumaepatula* (Tan et al., 2020). The QTLs were located in the substitution segments of SSSLs in the HJX74 genetic background. In total, twenty-one SSSLs carrying the 11 QTLs were used in this study, 10 of which were used as parents to develop pyramiding lines (Supplementary Table 1, Supplementary Figure 1).

### Field Experiments

All plant materials were planted in the farm of South China Agricultural University, Guangzhou (23°07′N, 113°15′E). The materials were planted in 2015–2020, two cropping seasons per year. The first cropping season (FCS) was from late February to mid-July, and the second cropping season (SCS) was from late July to mid-November. Germinated seeds were sown in a seedling bed, and seedlings were transplanted to the paddy field in a single seedling. Field cultivation and controlling of diseases and insect pests were followed by conventional methods in the local area.

### Genotyping of the QTLs for SER

All markers in the substitution segments carrying QTLs for SER were selected from the previous QTL mapping studies. The substitution segments and the QTLs for SER in SSSLs and pyramiding lines were detected following the previous studies (Tan et al., 2020, 2021, 2022). To distinguish different loci with the same name and to show the allelic origin of the QTLs, the first three letters of the donor species name were added to the suffix of the QTL names (Supplementary Table 1, Supplementary Figure 1).

### Phenotyping and Statistical Analysis

The SER and agronomic traits were investigated following the previous studies (Tan et al., 2020, 2021, 2022). For the statistical analysis, data of percentages were converted to the arcsine square root. The student's *t*-test was used to compare the two sets of data. Dunnett's *t*-test was used for multiple group comparison with the control group. The least significance range (LSR) was used for the multiple range test among multiple groups (Duncan, 1955). SPSS statistics 23.0 and OriginPro 9.0 were used for the data analysis and figure making (https://www.originlab.com).

### Estimation of Additive Effects and Epistatic Effects of QTLs

The additive effect of a QTL for SER was defined as the genotypic value of the homozygous genotype of a QTL in the HJX74 genetic background. Therefore, the additive effect of each locus was the difference in mean values of SER between HJX74 and an SSSL carrying a target QTL, and the additive effects of two- or multiple-locus genotypes were the difference in mean values of SER between HJX74 and pyramiding lines carrying two or multiple QTLs in the HJX74 genetic background. Epistasis of additive by additive interactions among QTLs for SER in 2- to 6-QTL lines was detected using homozygous genotypes. Epistatic effects among QTLs were estimated by the formula, $i=\left(P_{n}-P_{0}\right)-\overset{n}{\underset{i=1}{\sum }}\left(a_{i}\right)$, where *i* is an epistasis among the pyramided QTLs, *P*_*n*_ is a phenotype of a pyramiding line harboring *n* of QTLs, *P*_0_ is a phenotype of HJX74, *a*_*i*_ (1 ≤ *i* ≤ *n*) is an additive effect of a single QTL at the *i*th QTL. Epistatic effects among QTLs in pyramiding lines were tested in Student's *t-*test under null hypothesis (H_0_) *i* = 0.

## Results

### Genotypes in the Lines With 1- to 6-QTLs for SER

In total, eleven QTLs for SER were used to develop QTL-pyramiding lines. The QTLs were located in the substitution segments of SSSLs with the HJX74 genetic background. Three SSSLs, namely, A35, A42, and A88, each carried two SER-QTLs in a substitution segment, while the other 18 SSSLs all carried one SER-QTL in their substitution segments (Supplementary Table 1, Supplementary Figure 1). The 1-QTL lines (1QLs), which carried 1 QTL for SER in a substitution segment, were mutually crossed to develop 2-QTL lines (2QLs). The 2QLs were crossed with 1QLs to develop 3-QTL lines (3QLs) or crossed with other 2QLs to develop 4-QTL lines (4QLs). In this way, 5-QTL lines (5QLs) and 6-QTL lines (6QLs) were developed (Supplementary Figure 2).

A total of 29 QTL-pyramiding lines were developed from the SSSLs, which carried different combinations of QTLs for SER in the HJX74 genetic background (Table 1, Supplementary Table 2), including five 2QLs (Supplementary Figure 3), ten 3QLs (Supplementary Figure 4), seven 4QLs (Supplementary Figure 5), five 5QLs (Supplementary Figure 6), and two 6QLs (Supplementary Figure 7).

**Table 1:** SER-QTL combinations in pyramiding lines.

		**QTL**										
**Group**	**Pyramiding line**	***qSER1a-gla***	***qSER1b-gla***	***qSER1b-glu***	***qSER2a-sat***	***qSER2b-sat***	***qSER3a-sat***	***qSER3b-sat***	***qSER3b-glu***	***qSER5-glu***	***qSER8b-gla***	***qSER12-gla***
2-QTL line	A35	–	–	–	+	+	–	–	–	–	–	–
	A88	–	–	–	–	–	+	+	–	–	–	–
	2QL-1	–	+	–	–	–	–	–	–	–	–	+
	2QL-2	–	+	–	–	–	–	–	–	–	+	–
	2QL-3	–	–	–	–	–	–	–	–	–	+	+
	2QL-4	–	–	–	–	–	+	–	–	–	+	–
	2QL-5	–	–	–	–	–	+	–	–	–	–	+
3-QTL line	3QL-1	+	+	–	–	–	–	–	–	–	–	+
	3QL-2	+	+	–	–	–	–	–	–	–	+	–
	3QL-3	–	+	–	–	–	–	–	–	–	+	+
	3QL-4	+	–	–	–	–	–	–	–	–	+	+
	3QL-5	–	+	–	–	–	+	+	–	–	–	–
	3QL-6	–	–	–	–	–	+	+	–	–	+	–
	3QL-7	–	–	–	–	–	+	–	+	–	+	–
	3QL-8	–	–	+	–	–	+	–	–	–	+	–
	3QL-9	–	–	–	–	–	+	–	–	+	+	–
	3QL-10	–	–	–	+	+	+	–	–	–	–	–
4-QTL line	4QL-1	–	+	–	+	+	–	–	–	–	–	+
	4QL-2	–	+	–	+	+	–	–	–	–	+	–
	4QL-3	–	–	–	+	+	–	–	–	–	+	+
	4QL-4	–	+	–	–	–	+	+	–	–	–	+
	4QL-5	–	–	–	–	–	+	+	–	–	+	+
	4QL-6	–	+	–	–	–	+	+	–	–	+	–
	4QL-7	+	–	–	–	–	+	+	–	–	–	+
5-QTL line	5QL-1	–	+	–	–	–	+	+	–	–	+	+
	5QL-2	–	–	–	+	+	+	+	–	–	–	+
	5QL-3	–	–	–	–	–	+	+	+	+	+	–
	5QL-4	–	+	–	–	–	+	+	–	+	+	–
	5QL-5	–	–	–	+	+	+	+	–	–	+	–
6-QTL line	6QL-1	–	–	–	+	+	+	+	–	+	+	–
	6QL-2	–	+	–	+	+	+	+	–	–	+	–

*“+,” Presence; “–,” Absence; SER, stigma exsertion rate. A35 and A88 are the single-segment substitution lines (SSSLs) carrying two QTLs for SER in the substitution segments*.

### Phenotypes in the Lines With 1- to 6-QTLs for SER

**Figure 1:**
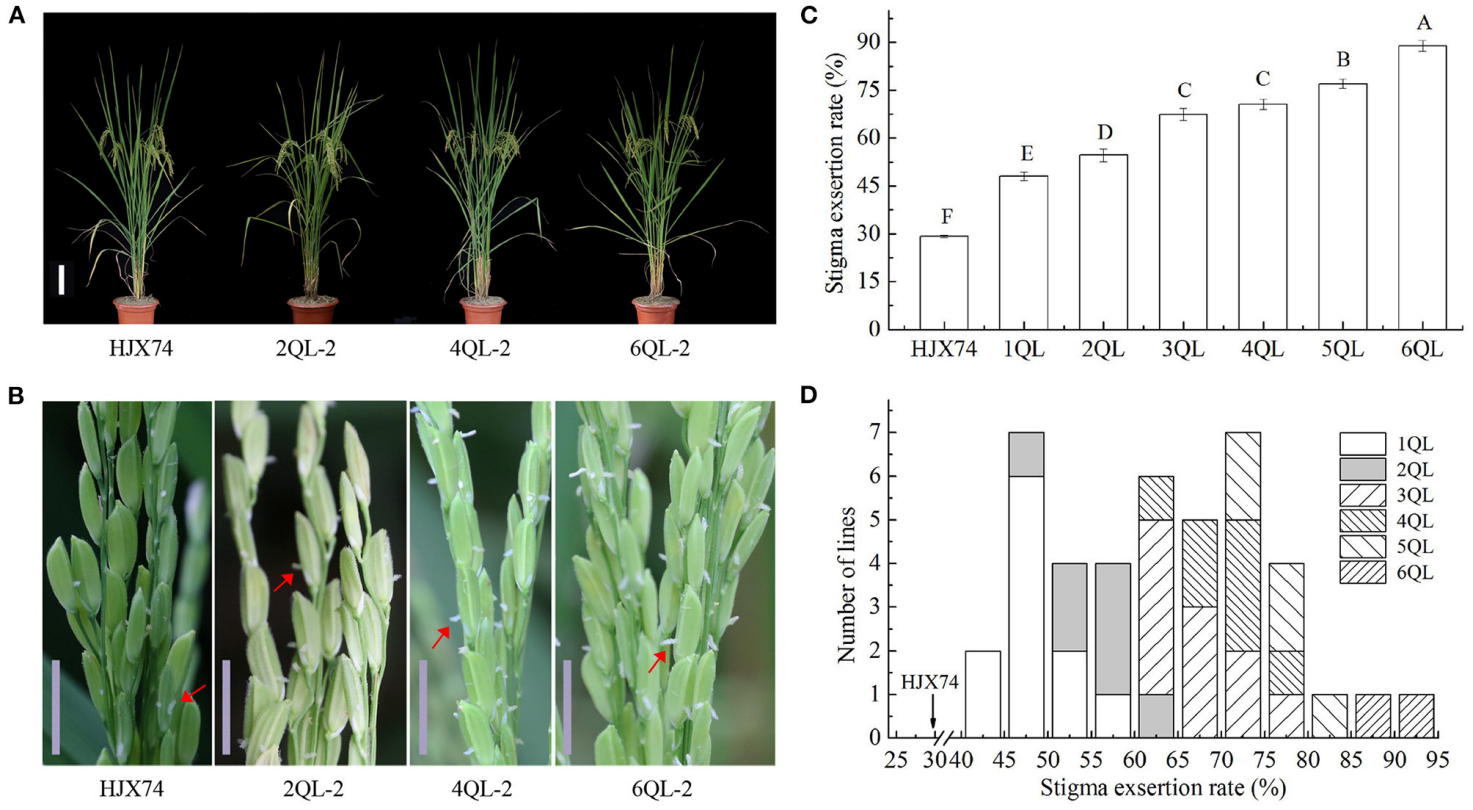
Stigma exsertion rate (SER) of 1- to 6-QTL lines. **(A)** Plant types in the 2QL-2, 4QL-2, and 6QL-2 lines and HJX74. Scale bar, 15 cm. **(B)** The phenotypes of stigma exsertion in the 2QL-2, 4QL-2, and 6QL-2 lines and HJX74. Scale bar, 1 cm. The red arrows point to the exposed stigma. **(C)** SER of 1- to 6-QTL lines. HJX74 as a control. Data are shown as the mean ± S.E. of three cropping seasons. Capital letters indicate the significance of differences at the 1% levels. **(D)** Frequency distributions of SER in 1- to 6-QTL lines.

The SER in the lines with 1- to 6-QTLs were investigated in three cropping seasons (Figures 1A–D). The average SER for 1-QTL lines (1QLs) was 48.1%, ranging from 42.5 to 56.5%, which was significantly higher than the 29.2% of HJX74 (Figures 1C,D, Supplementary Table 3). In seven 2QLs including two SSSLs each carrying 2 QTLs in a single substitution segment and five QTL-pyramiding lines, the average SER was 54.6%, ranging from 46.9 to 63.2%, which was 6.5% higher than the SER in 1QLs. In ten 3QLs, the average SER was 67.4%, ranging from 60.0 to 78.1%, which was 12.8% higher than the SER in 2QLs. In seven 4QLs, the average SER was 70.6%, ranging from 63.4 to 76.5%, which was 3.2% higher than the SER in 3QLs. In five 5QLs, the average SER was 77.1%, ranging from 73.3 to 80.9%, which was 6.5% higher than the SER in 4QLs. In two 6QLs, the average SER was 88.8%, ranging from 86.0 to 91.7%, which was 11.7% higher than the SER in 5QLs (Figures 1C,D, Supplementary Table 4). These results showed that SERs increased with the increase of QTLs in pyramiding lines.

Phenotypes of eight agronomic traits in 2- to 6-QTL lines were investigated. Most of the traits in these lines, such as heading date, plant height, grain number per panicle, panicle length, and seed setting rate, were not significantly different from those of HJX74. In grain length, 13 lines were significantly different from HJX74, while the other 18 lines were not significantly different. In grain width, 14 lines were significantly different from HJX74, while the other 17 lines were not significantly different (Supplementary Table 5). These results showed that the pyramiding lines had a similar genetic background to HJX74, except for the genotype of SER. The grain size, including grain length and grain width, had no significant effect on SER.

### Additive Effects of QTLs for SER in 1- to 6-QTL Lines

**Figure 2:**
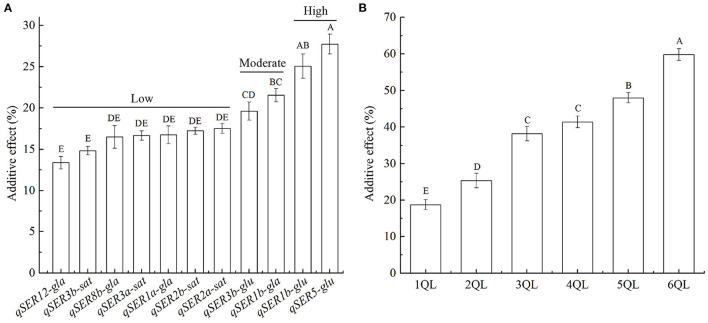
Additive effects of QTLs on stigma exsertion rate (SER). **(A)** Additive effects of 11 QTLs on SER. **(B)** Additive effects of QTL combinations on SER in 1- to 6-QTL lines. Data are shown as the mean ± S.E. of three cropping seasons. Capital letters indicate the significance of differences at the 1% levels.

The additive effects of 11 QTLs on SER varied greatly. The average additive effect of 11 QTLs on SER was 18.9%, ranging from 13.3 to 27.3%. Based on the additive effects, 11 QTLs could be divided into three levels. There were 7 QTLs at the low-effect level with the average additive effect of 16.2%, ranging from 13.3 to 17.5%. There were two QTLs at the moderate-effect level with the average additive effect of 20.8%, ranging from 20.2 to 21.4%. At the high-effect level, the additive effects of two QTLs were 25.5 and 27.3%, respectively, and 26.4% on average (Figure 2A, Supplementary Table 6). The 11 QTLs were from three donors. Four QTLs from *O*. *sativa* showed the low additive effects with the average additive effect of 16.6%, ranging from 14.8 to 17.5%. The average additive effect of four QTLs from *O*. *glaberrima* was 17.1%, ranging from 13.3 to 21.4% with three low-effect QTLs and one moderate-effect QTL. The average additive effect of three QTLs from *O*. *glumaepatula* was 24.3%, ranging from 20.2 to 27.3% with two high-effect QTLs and one moderate-effect QTL (Figure 2A). The magnitude of additive effects of QTLs for SER was consistent with the evolutionary levels of species.

Additive effects of 2- to 6-QTLs were calculated from the SER in 2- to 6-QTL lines. The average additive effect of 2-QTL combinations on SER was 25.4% in seven 2QLs, ranging from 17.7 to 34.1%. The average additive effect of 3-QTL combinations on SER was 38.3% in ten 3QLs, ranging from 30.9 to 48.9%. The average additive effect of 4-QTL combinations on SER was 41.4% in seven 4QLs, ranging from 34.3 to 47.3%. The average additive effect of 5-QTL combinations on SER was 47.9% in five 5QLs, ranging from 44.2 to 51.8%. The average additive effect of 6-QTL combinations on SER was 59.7% in two 6QLs, ranging from 56.8 to 62.6% (Figure 2B, Supplementary Table 7). These results showed that the total additive effects of QTLs increased with increasing QTLs in 1- to 6-QTL lines.

### Epistasis of Additive by Additive Interaction Among QTLs on SER in 2- to 6-QTL Lines

**Figure 3:**
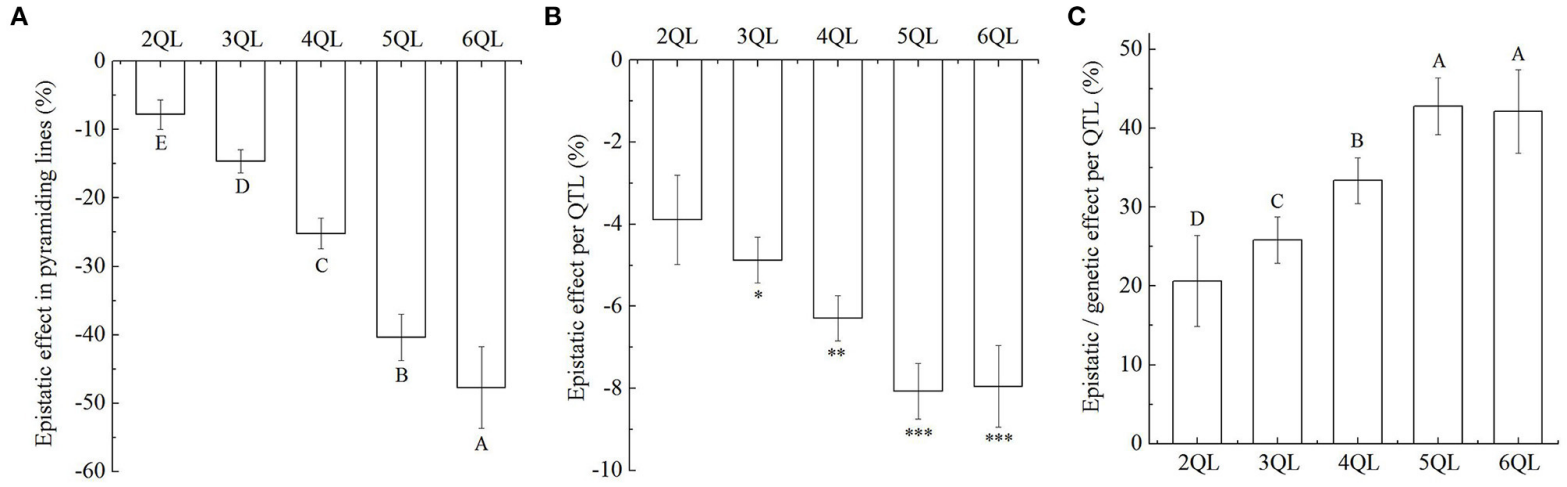
Epistatic effects of QTLs on stigma exsertion rate (SER). **(A)** The total epistatic effects of QTL combinations on SER in 2- to 6-QTL lines. **(B)** The average epistatic effects per QTL on SER in 2- to 6-QTL lines. *P ≤ 0.05; **P ≤ 0.01; ***P ≤ 0.001. **(C)** Percentage of epistatic effects in genetic effects for each QTL in 2- to 6-QTL lines. Data are shown as the mean ± S.E. of three cropping seasons. Capital letters indicate the significance of differences at the 1% levels.

Although the additive effects of QTLs on SER were increasing in 2- to 6-QTL lines, they were generally below the expected values (Figure 2B, Supplementary Table 4). It suggested that there were epistatic effects of QTLs in the 2- to 6-QTL lines. Among the seven 2QLs, the average epistatic effect per QTL combination was −7.8%, ranging from 0.9 to −14.1%, where the average epistatic effect per QTL was −3.9%, but the epistatic effect was not significant. Among the ten 3QLs, the average epistatic effect per QTL combination was −14.6%, ranging from −3.9 to −21.4%, where the average epistatic effect per QTL was −4.9%, and the epistatic effect was significant at the 0.05 level. Among the seven 4QLs, the average epistatic effect per QTL combination was −25.2%, ranging from −17.0 to −31.2%, where the average epistatic effect per QTL was −6.3%, and the epistatic effect was significant at the 0.01 level. Among the five 5QLs, the average epistatic effect per QTL combination was −40.4%, ranging from −32.4 to −49.0%, where the average epistatic effect per QTL was −8.1%, and the epistatic effect was significant at the 0.001 level. Among the two 6QLs, the average epistatic effect per QTL combination was −47.7%, ranging from −41.7 to −53.7%, where the average epistatic effect per QTL was −8.0%, and the epistatic effect was significant at the 0.001 level (Figures 3A,B, Supplementary Table 8). These results showed that the additive effect of multiple QTLs was smaller than the sum of the additive effects of the corresponding single QTL. The epistatic interactions between QTLs in pyramiding lines reduced the total additive effect of QTL combinations. The epistatic effects of QTLs increased with increasing QTLs in 2- to 6-QTL lines. Therefore, the epistasis was less-than-additive or negative effect.

In 2- to 6-QTL lines, the percentage of epistatic effects in genetic effects for each QTL were 20.7, 26.0, 33.4, 42.9, and 42.4%, respectively (Figure 3C). The results showed that although there was epistasis, additivity was the major component of QTL interaction.

### QTL *qSER3a-sat* Showed a Minor Epistasis Effect

**Figure 4:**
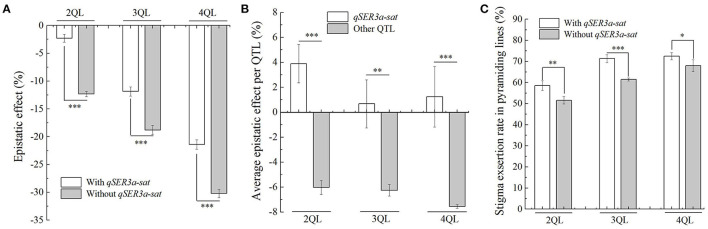
Epistatic effect of *qSER3a-sat* on stigma exsertion rate (SER). **(A)** Epistatic effects of the QTLs in the 2- to 4-QTL lines with and without *qSER3a-sat*. **(B)** Epistatic effects of a single QTL of *qSER3a-sat* and other QTLs in 2- to 4-QTL lines. **(C)** SER in the 2- to 4-QTL lines with and without *qSER3a-sat*. Data are shown as the mean ± S.E. of three cropping seasons. *P ≤ 0.05; **P ≤ 0.01; ***P ≤ 0.001.

In the 2- to 4-QTL lines, there were two genotypes with and without *qSER3a-sat*. In 2QLs, the average epistatic effect of three lines with *qSER3a-sat* was −2.1%, while that of four lines without *qSER3a-sat* was −12.0%. In 3QLs, the average epistatic effect of six lines with *qSER3a-sat* was −11.9%, while that of four lines without *qSER3a-sat* was −18.8%. In 4QLs, the average epistatic effect of four lines with *qSER3a-sat* was −21.4%, while that of three lines without *qSER3a-sat* was −30.4%. In the three sets of QTL lines, the average epistatic effects in the lines with *qSER3a-sat* were significantly less than those without *qSER3a-sat* (Figure 4A, Supplementary Tables 8, 9). In 2- to 4-QTL lines, the epistatic effects of *qSER3a-sat* in the *qSER3a-sat* groups were 3.9, 0.7, and 1.4%, respectively, while the average epistatic effects per QTL in the *qSER3a-sat*-free groups were −6.0, −6.3, and −7.6%, respectively (Figure 4B, Supplementary Table 9). Differing from other QTLs, the epistatic effects of *qSER3a-sat* on SER were minor and positive. As a result, the lines with *qSER3a-sat* had greater SER than those without *qSER3a-sat*. In 2QLs, the average SER of the lines with *qSER3a-sat* was 58.6%, while that of the lines without *qSER3a-sat* was 51.6%. In 3QLs, the average SER of the lines with *qSER3a-sat* was 71.4%, while that of the lines without *qSER3a-sat* was 61.5%. In 4QLs, the average SER of the lines with *qSER3a-sat* was 72.5%, while that of the lines without *qSER3a-sat* was 68.0% (Figure 4C, Supplementary Table 4). These results showed that *qSER3a-sat* significantly increased SER than other QTLs in the pyramiding lines due to its minor epistasis effect.

## Discussion

### The Reduced Additive Effect of QTLs on SER May Be Responsible for the Degradation of Outcrossing Ability in Cultivated Rice

The emergence of cultivated crops is the result of natural and artificial selection. During the domestication of cultivated rice, the selection of varieties led to homozygosity of genotypes. At the same time, genotypic homozygosity reinforced the inbreeding of varieties, leading to a gradual decline in their outcrossing ability. Therefore, the degradation of outcrossing ability is an inevitable outcome of the domestication of cultivated rice. Carter et al. (2005) studied the effects of different forms of epistasis on the response to directional selection using a multilinear epistatic model and showed that the main factor determining the evolution of the additive variance, and thus the evolvability, is directional epistasis. Doust et al. (2014) argued that domestication is a multifaceted evolutionary process, involving changes in individual genes, gene interactions, and emergent phenotypes, and found that alleles favored during domestication tend to have larger phenotypic effects and are relatively insensitive to genetic background and environmental influences compared with wild progenitor alleles. In this study, the additive effects of 11 QTLs could be divided into three levels. The four QTLs from *O*. *sativa*, an Asian cultivated rice, were the low additive effects. *O*. *glaberrima*, the recently domesticated African cultivated rice, had three low-effect QTLs and one moderate-effect QTL. Among the three QTLs from *O*. *glumaepatula*, a wild species, two were high-effect QTLs and one was moderate-effect QTL (Figure 2A). The magnitude of additive effects of QTLs for SER was consistent with the evolutionary levels of species. In the process of domestication, the additive effects of QTLs on SER were reduced in cultivated rice. Therefore, the reduction in the additive effect of QTLs on SER during domestication may be an important factor in the degradation of the outcrossing ability of cultivated rice.

### The Epistasis of Additive by Additive Interaction Between QTLs on SER Was Less-Than-Additive

Mapping epistatic interactions is experimentally, statistically, and computationally challenging. The classical populations used to map QTLs are poorly efficient to detect epistasis. The use of secondary mapping populations, such as chromosome segment substitution lines (CSSLs), introgression lines (ILs), and NILs, in which a region containing the QTL is introgressed into the isogenic background of one of the parental lines, and the QTLs are narrowed down to a small genomic interval by recombination in successive generations, facilitates the analysis of epistasis between naturally occurring variants (Causse et al., 2007; Mackay, 2014). Eshed and Zamir (1996) analyzed interactions between individual *Lycopersicon pennellii* chromosome segments introgressed into an otherwise homogeneous genetic background of *L. esculentum* in a half diallele scheme. Of the 180 tested interactions, 28% were epistatic on both linear and geometric scales, and the detected epistasis was predominately less-than-additive. Although the frequency of epistasis was high, additivity was the major component in the interaction of pairs of QTLs. Kroymann and Mitchell-Olds (2005) reported that a small chromosome interval with no effect on the growth rate of *A. thaliana* NILs contained two epistatically interacting QTLs affecting growth, for one of which the effect on growth was in opposite directions in the different genetic backgrounds. Secondary mapping populations were also used to detect epistasis of QTLs in tomato (Causse et al., 2007) and rice (Uwatoko et al., 2008; Chen et al., 2014; Qin et al., 2015; Yang et al., 2018; Misra et al., 2021). In this study, we used pyramiding lines with 2- to 6-QTLs for SER developed by SSSLs to detect epistasis at two or more loci. Due to the SSSLs and pyramiding lines being homozygous genotypes in the HJX74 genetic background, only the epistasis of additive by additive interaction was detected. The results showed that while the epistasis of QTLs increased with increasing QTLs in 2- to 6-QTL lines, the total additive effects of QTLs increased (Figures 2B, 3). These results indicated that the epistatic interactions of the QTLs only changed the magnitude of effects, in which the phenotype of one locus is suppressed by genotypes at the other locus. Therefore, the epistasis of additive by additive interaction among QTLs in 2- to 6-QTL lines was less-than-additive or negative effect. The results also suggest that detecting epistasis of additive-additive interactions in pyramiding lines developed from SSSLs of the same genetic background is a simpler and more effective scheme.

### The High-SER Trait Lost in the Domestication of Cultivated Rice Can Be Reconstructed

In the past decades, great progress has been made in the heterosis utilization of hybrid rice (Yuan, 2017; Zhang, 2022). Improving the outcrossing ability of MSLs is very important for hybrid rice seed production. In the past two decades, a larger number of QTLs controlling SER and related traits have been identified on 12 chromosomes of the rice genome from various genetic resources (Marathi and Jena, 2015; Zhou et al., 2017a; Liu et al., 2019). Recently, eighteen QTLs for SER from *O*. *sativa, O*. *glaberrima*, and *O*. *glumaepatula* were detected in the SSSLs in the HJX74 genetic background (Tan et al., 2020, 2021, 2022). It laid a foundation for improving the outcrossing ability of MSLs of cultivated rice using the QTLs for SER. Most MSLs and wild rice were found to have the SER of 70% or more (Virmani and Athwal, 1973; Ying and Zhang, 1989; Uga et al., 2003b; Li et al., 2014; Lou et al., 2014; Guo et al., 2017; Zou et al., 2020). In this study, we found that despite the presence of widespread epistasis, additivity remained a major component of QTL interactions, which resulted in an increase in the SER with increasing QTLs in the pyramiding lines. When carrying 5–6 QTLs, the pyramiding lines had the SER level of most MSLs and wild rice (Figure 1). In addition, it was found that *qSER3a-sat* had a minor epistasis, which was more effective for improving the SER (Figure 4). Our study suggests that it is possible to reconstruct the high-SER trait, which was lost during the domestication of cultivated rice. The understanding of the genetic architecture of SER in rice lays the foundation for reconstructing the high-SER trait in MSL breeding.

## Data Availability Statement

The original contributions presented in the study are included in the article/Supplementary Material, further inquiries can be directed to the corresponding authors.

## Author Contributions

GZ and SW designed and supervised the work. QT and SB performed most of the experiments and prepared the experimental data. GC, ZY, ZC, WY, PZ, SL, LX, and SC conducted a part of the experiments. HZ, GL, and ZL prepared the experimental materials and assisted in the experiments. GZ analyzed the data and wrote the manuscript. All authors read and approved the final manuscript.

## Funding

This work was supported by grants from the Major Program of Transgenic New Variety Breeding of China (2014ZX08009-037B) and from the National Natural Science Foundation of China (91435207 and 91735304).

## Conflict of Interest

The authors declare that the research was conducted in the absence of any commercial or financial relationships that could be construed as a potential conflict of interest.

## Publisher's Note

All claims expressed in this article are solely those of the authors and do not necessarily represent those of their affiliated organizations, or those of the publisher, the editors and the reviewers. Any product that may be evaluated in this article, or claim that may be made by its manufacturer, is not guaranteed or endorsed by the publisher.
