# Supplementary material for: Reconstruction of the High Stigma Exsertion Rate Trait in Rice by Pyramiding Multiple QTLs
Source: Front Plant Sci. 2022 Jun 7;13:921700. doi: 10.3389/fpls.2022.921700 (PMC9209754; doi:10.3389/fpls.2022.921700)
Supplement: Supplementary file 1 [file Data_Sheet_1.PDF]

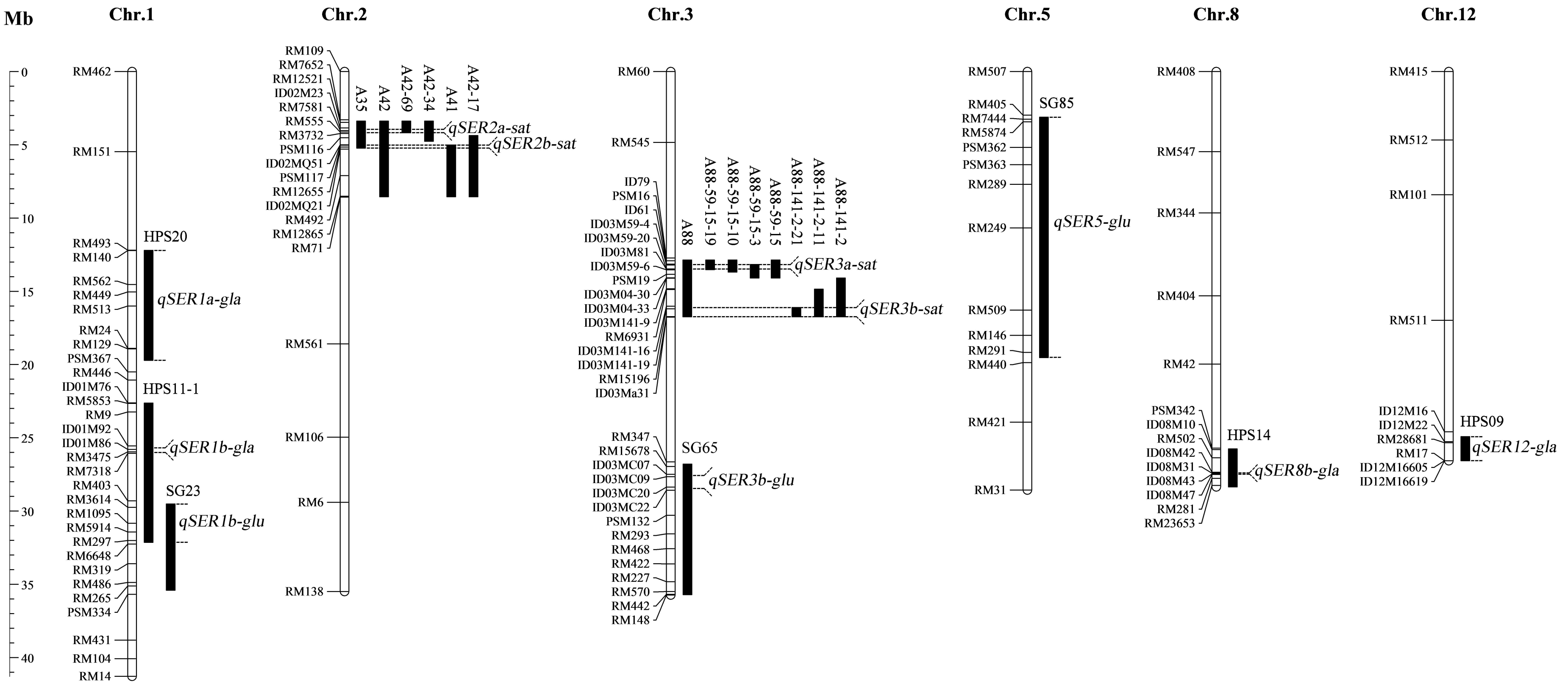

Supplementary Figure 1 QTLs for SER and their position in the substitution segments in SSSLs.

SER, stigma exsertion rate. SSSL, single-segment substitution line.

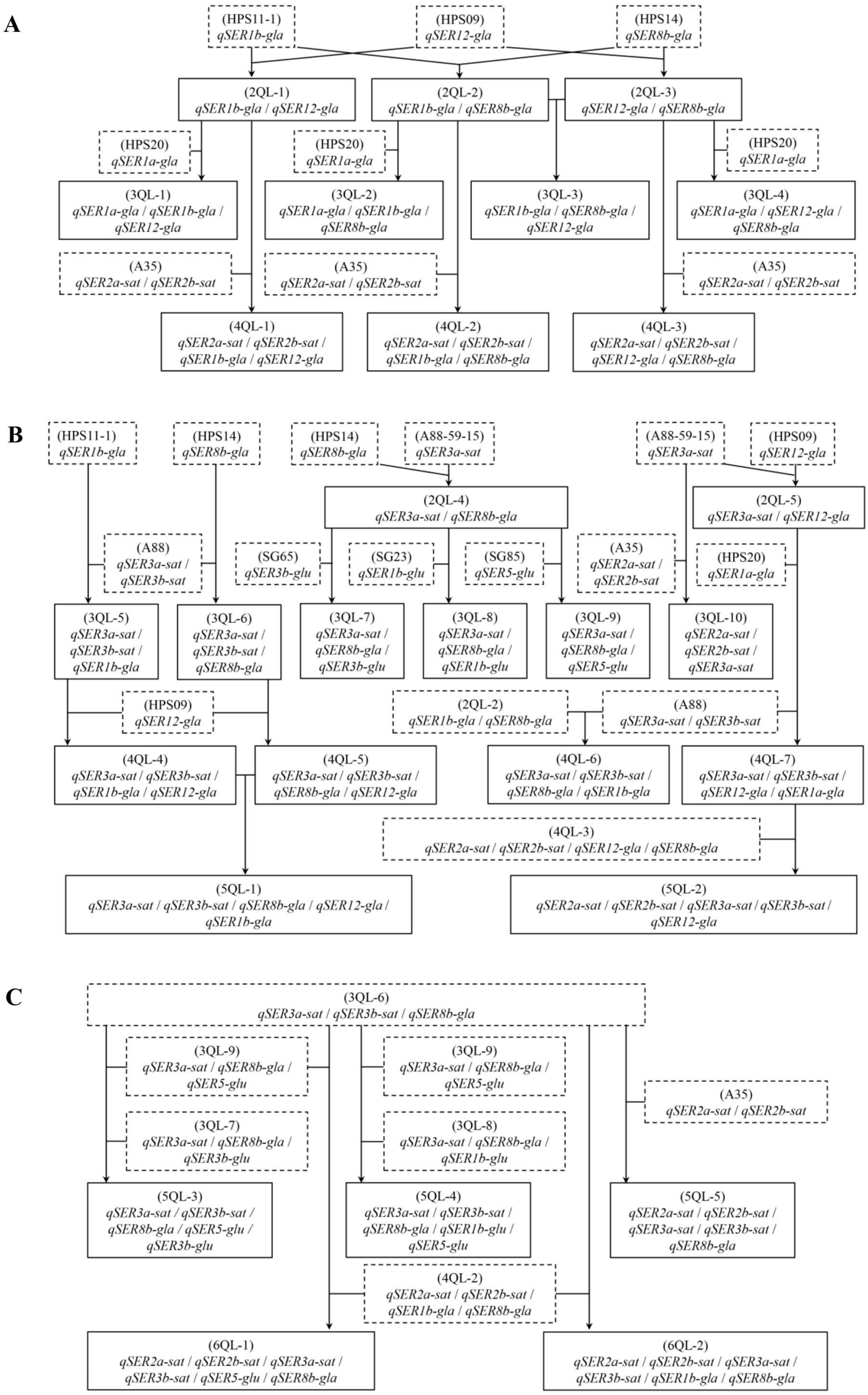

Supplementary Figure 2 Development of pyramiding lines with different SER-QTL combinations. (**A**) Development of partial 2-QTL lines (2QLs), 3QLs and 4QLs. (**B**) Development of partial 2QLs, 3QLs, 4QLs and 5QLs. (**C**) Development of partial 5QLs and 6QLs. The names in dotted boxes are the parents of pyramiding lines. The names in solid boxes are the developed pyramiding lines.

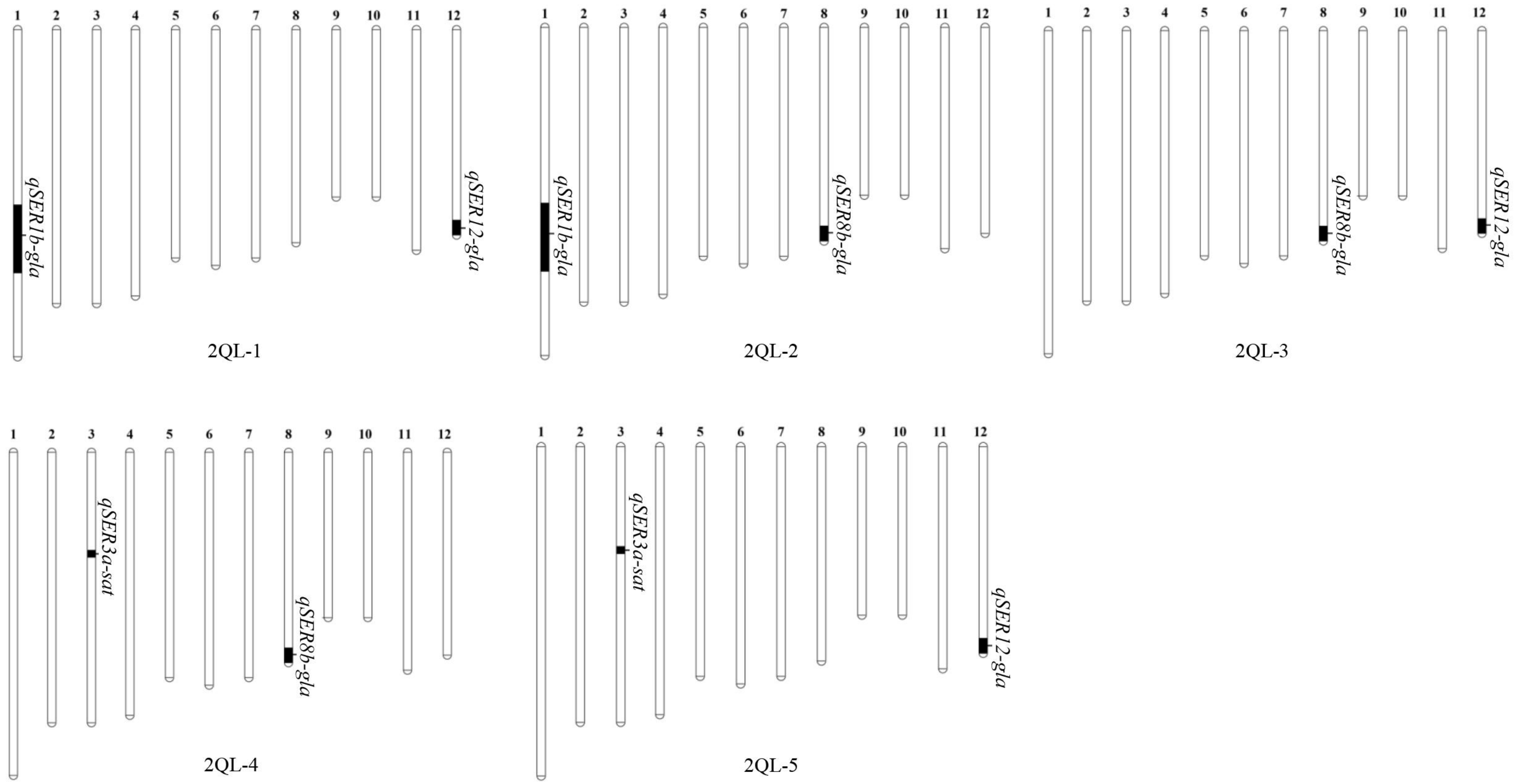

Supplementary Figure 3 QTLs for SER and their substitution segments in 2-QTL lines (2QLs).

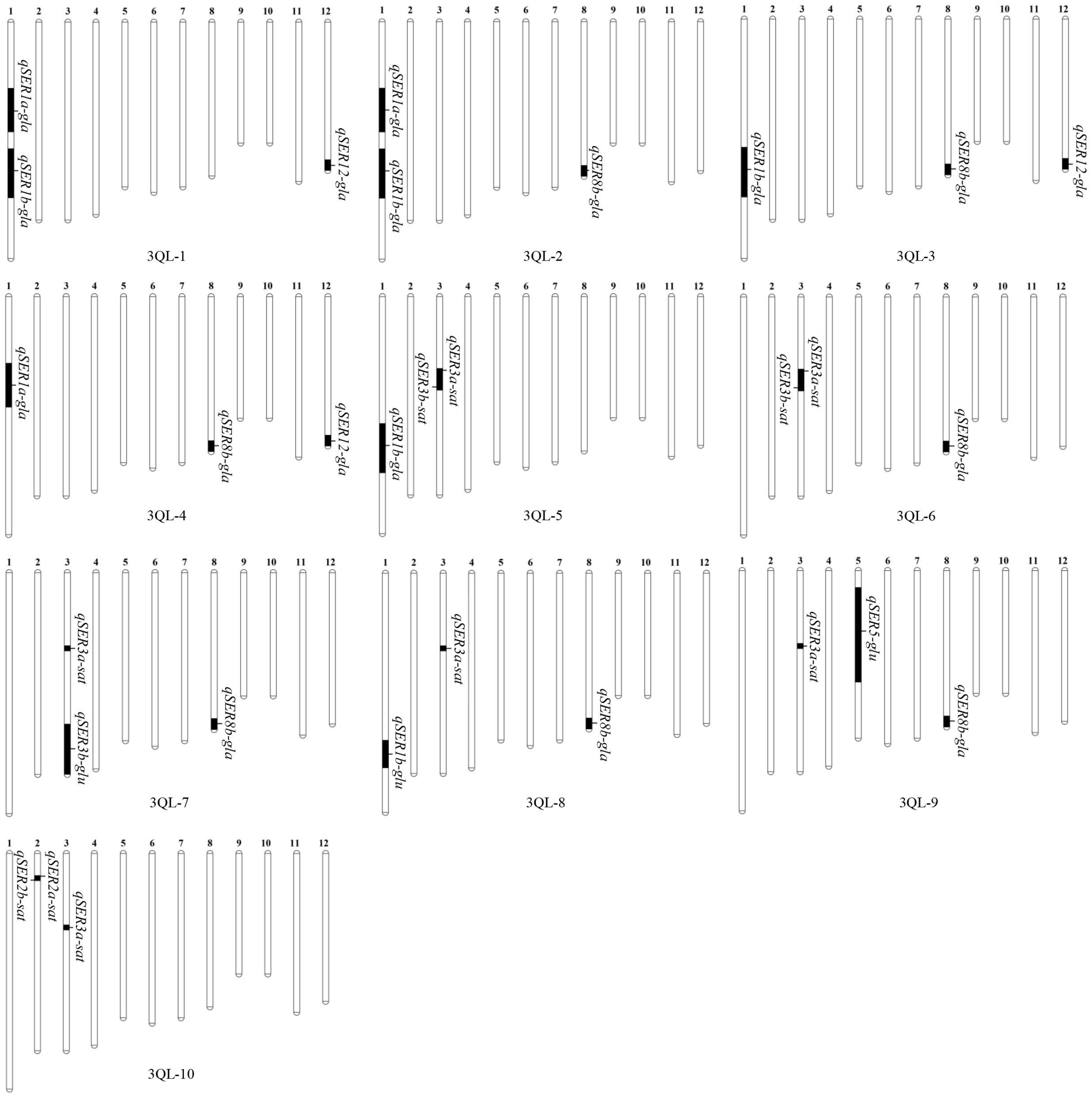

Supplementary Figure 4 QTLs for SER and their substitution segments in 3-QTL lines (3QLs).

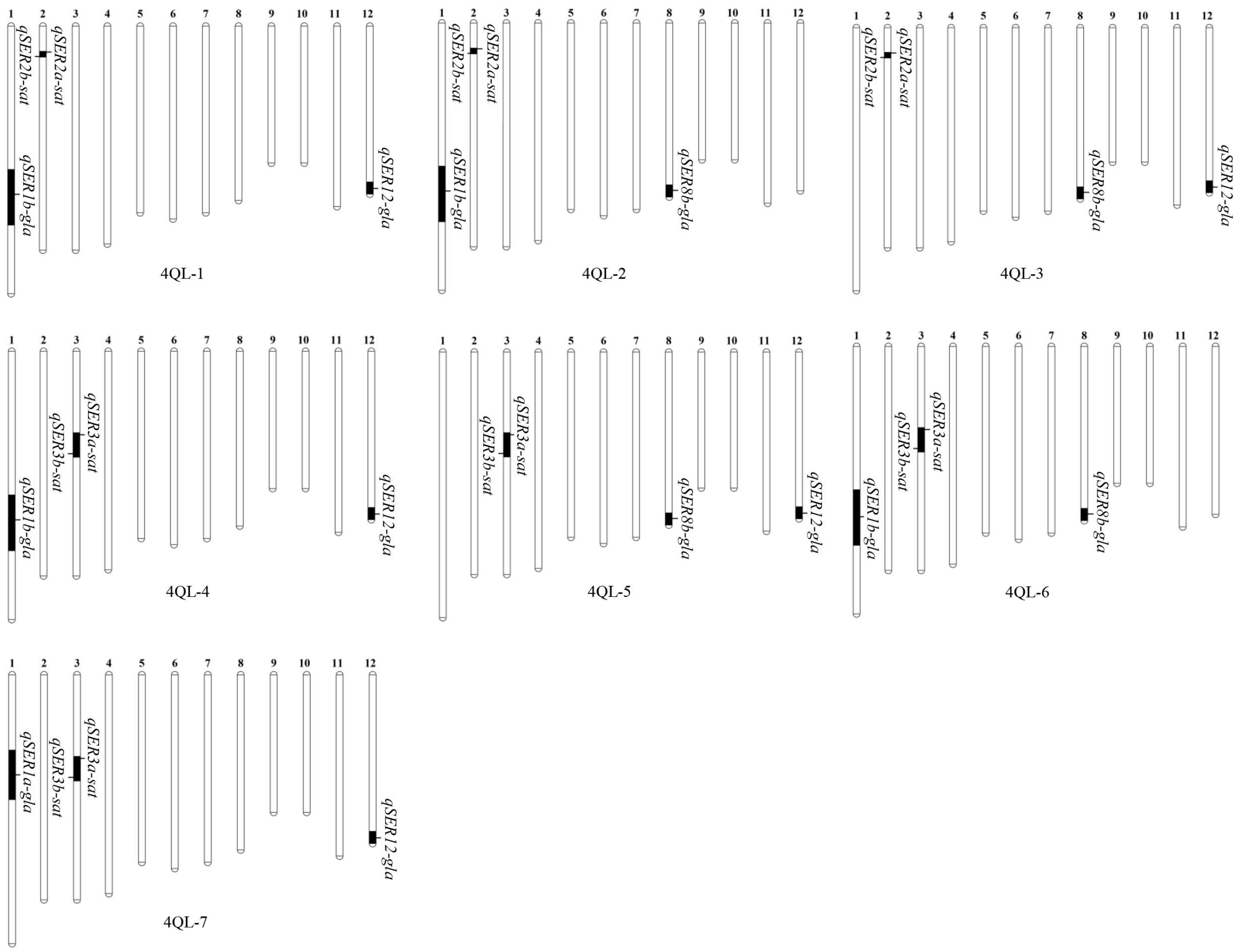

Supplementary Figure 5 QTLs for SER and their substitution segments in 4-QTL lines (4QLs).

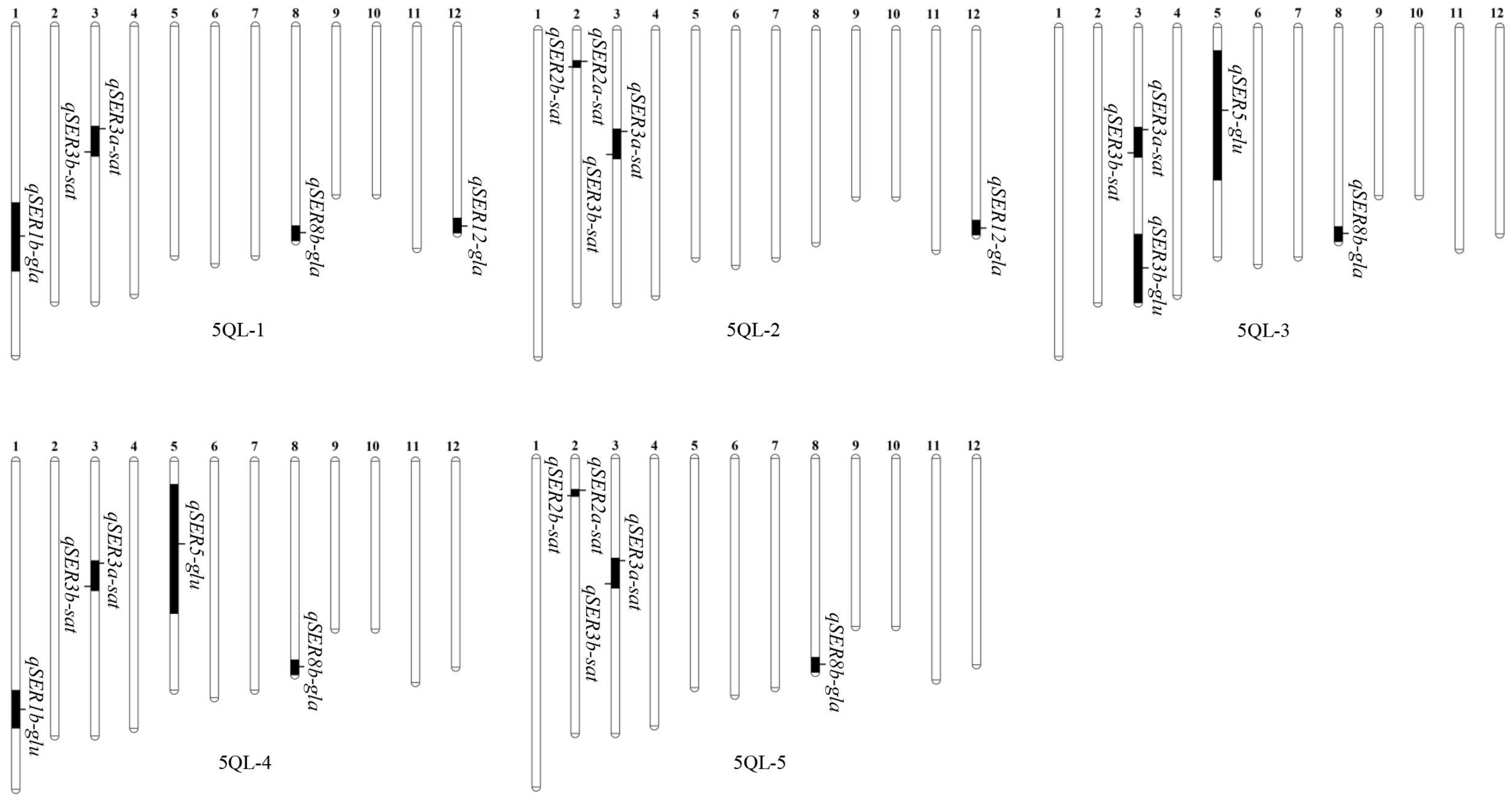

Supplementary Figure 6 QTLs for SER and their substitution segments in 5-QTL lines (5QLs).

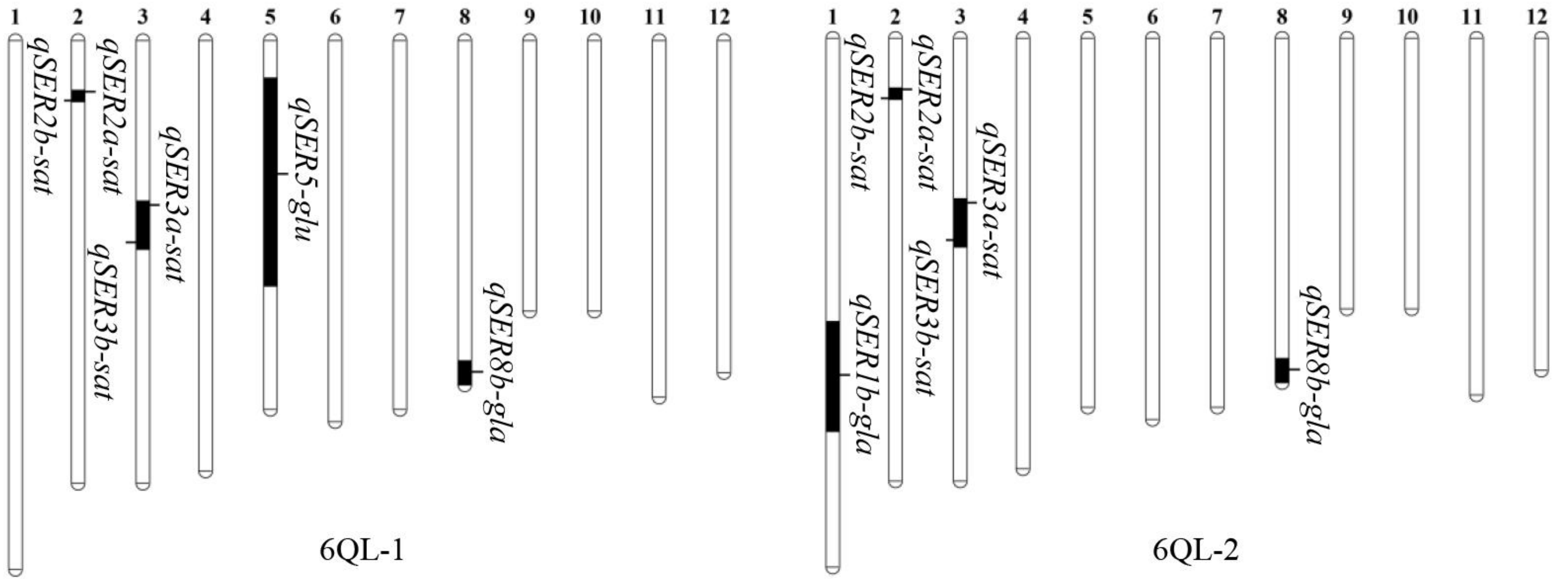

Supplementary Figure 7 QTLs for SER and their substitution segments in 6-QTL lines (6QLs).
